# D-allose reprogrammes the mitophagy–mtDNA–cGAS–STING–ADM2 axis to restore nucleus pulposus cell homeostasis in intervertebral disc degeneration

**DOI:** 10.1038/s12276-026-01844-7

**Published:** 2026-10-01

**Authors:** Zhuoyang Zhao, Jiamin Li, Linchuan Lei, Yanting Chen, Zhe Li, Yuhua Zhang, Zhaomin Zheng, Hua Wang, Fan Chen, Jianru Wang

**Affiliations:** 1 Department of Spine Surgery, The First Affiliated Hospital of Sun Yat-sen University, Guangzhou, 510080 China; 2 Guangdong Province Key Laboratory of Orthopedics and Traumatology, Guangzhou, 510080 PR China; 3 Laboratory of General Surgery, The First Affiliated Hospital, Sun Yat-sen University, Guangzhou, 510080 China; 4 Guangdong Provincial Key Laboratory of Advanced Biomaterials, Department of Biomedical Engineering, Southern University of Science and Technology, Shenzhen, 518055 People’s Republic of China; 5 Kinesphere Lab, Irvine High School, 4321 Walnut Ave, Irvine, California 92604 USA; 6 Pain Research Center, Sun Yat-Sen University, Guangzhou, 510080 China; 7 Shenzhen Key Laboratory of Spine Surgery, Department of Spine Surgery, Peking University Shenzhen Hospital, Shenzhen, China

**Keywords:** Senescence, Apoptosis, Autophagy, Innate immunity, Biologics

## Abstract

Intervertebral disc degeneration (IVDD) is driven by progressive loss of nucleus pulposus cell (NPC) homeostasis, yet the stress-responsive mechanisms governing this process remain incompletely defined. Here, we identify a mitochondrial stress–innate immune signalling axis in which impaired mitophagy promotes mitochondrial DNA (mtDNA) leakage, aberrant activation of the cGAS–STING pathway, suppression of the cytoprotective peptide adrenomedullin 2 (ADM2) and consequent NPC senescence and apoptosis. Using integrated transcriptomic analyses, genetic models and human disc specimens, we establish ADM2 as a previously unrecognized downstream effector linking innate immune activation to disc cell fate control. Importantly, we demonstrate that D-allose, a naturally occurring rare sugar with an excellent safety profile, reprogrammes this pathogenic axis by restoring mitophagy, limiting cytosolic mtDNA accumulation, suppressing cGAS–STING signalling and preserving ADM2-dependent cytoprotection. In vivo, D-allose markedly attenuates IVDD, with genetic ablation of STING providing complementary evidence that suppression of this pathway confers robust protection against degeneration. Together, these findings define a mitophagy–mtDNA–cGAS–STING–ADM2 axis as a central regulator of IVDD and reveal D-allose as a physiologically compatible strategy for rebalancing mitochondrial stress signalling and restoring NPC homeostasis.

## Mitophagy and Immune Signalling Control Disc Cell Fate

Intervertebral disc degeneration (IVDD) is a major cause of back pain, driven by stress-induced disruptions in nucleus pulposus cell (NPC) homeostasis. This study identifies D-allose, a naturally occurring rare sugar, as a potential therapeutic agent that modulates the mitochondrial DNA–cGAS–STING–ADM2 axis to restore NPC function. Researchers used in vivo and in vitro models to explore the effects of D-allose on NPCs, showing that it restores mitophagy, reduces cytosolic mitochondrial DNA and suppresses cGAS–STING signalling. This leads to decreased NPC senescence and apoptosis, preserving disc integrity. The ability of D-allose to enhance ADM2 expression, a key downstream effector, highlights its protective role. These findings suggest that D-allose might be a promising intervention for IVDD, offering a safe, long-term treatment option. Future research should explore the pharmacokinetics and optimal administration strategies of D-allose to enhance its clinical applicability in treating IVDD and other degenerative conditions.

This summary was initially drafted using artificial intelligence, then revised and fact-checked by the author.

## Introduction

Intervertebral disc degeneration (IVDD) is a leading cause of low back pain and disability worldwide, imposing a substantial socioeconomic burden^1,2^. The intervertebral disc is an avascular fibrocartilaginous tissue in which nucleus pulposus cells (NPCs) are essential for maintaining extracellular matrix (ECM) homeostasis and biomechanical integrity^3,4^. Progressive NPC senescence and apoptosis disrupt matrix turnover and drive structural degeneration of the disc, ultimately leading to pain and functional impairment^5–8^. Despite growing insights into IVDD pathophysiology, effective disease-modifying therapies remain lacking^3,9^. Current clinical strategies largely focus on symptomatic relief rather than targeting the underlying mechanisms. Consequently, identifying safe, well-tolerated and mechanism-based interventions suitable for long-term modulation of NPC homeostasis remains an urgent unmet need.

Mitochondrial dysfunction accompanied by mitochondrial DNA (mtDNA) leakage is increasingly implicated in the pathogenesis of degenerative diseases^10^. Excessive accumulation of cytosolic mtDNA, acting as a damage-associated molecular pattern (DAMP), can trigger cellular senescence and apoptosis^11,12^. Mitophagy, the selective autophagic clearance of damaged mitochondria, has been identified as a critical mitochondrial quality control mechanism that limits mtDNA release^13^. Accordingly, impaired mitophagy has been associated with cytosolic mtDNA accumulation^14^. Despite growing interest in the role of mitophagy in IVDD^15,16^, the mechanistic link between mitophagy impairment, cytosolic mtDNA accumulation and disease progression remains incompletely characterized. Furthermore, therapeutic strategies aimed at restoring defective mitophagy to alleviate mtDNA-associated cellular stress in IVDD are still limited.

To sense and respond to aberrant cytoplasmic DNA, cells rely on innate immune surveillance pathways. Among these, the cyclic GMP–AMP synthase–stimulator of interferon genes (cGAS–STING) pathway functions as a principal sensor of cytoplasmic mtDNA and a key mediator of mtDNA-driven degenerative signalling^17^. However, despite these mechanistic insights, how this stress-responsive network can be therapeutically rebalanced remains unclear. Moreover, the endogenous downstream effectors that translate cGAS–STING activation into NPC fate decisions remain poorly defined, limiting the development of more targeted therapeutic strategies for IVDD.

Adrenomedullin 2 (ADM2), also known as intermedin, is a bioactive peptide belonging to the calcitonin gene-related peptide family. Previous studies have shown that ADM2 exerts cytoprotective effects by modulating cellular stress responses, including anti-apoptotic, anti-inflammatory and stress-adaptive functions^18–21^. Despite these protective properties, whether ADM2 participates in the regulation of NPC fate during IVDD and whether it acts downstream of cGAS–STING signalling remain unknown.

Given the lack of safe and sustainable therapeutic strategies for IVDD, naturally derived small molecules with favourable safety profiles represent an attractive alternative. Rare sugars have recently gained attention owing to their low toxicity and multiple physiological functions^22^. D-allose is a naturally occurring rare sugar characterized by ultra-low caloric content and negligible toxicity, and has been reported to exert anti-oxidative, anti-inflammatory and anti-apoptotic effects in diverse diseases^23–26^. These properties suggest a previously unrecognized therapeutic potential of D-allose in IVDD. However, whether D-allose can restore mitophagy, restrain aberrant mtDNA–cGAS–STING signalling and thereby influence downstream effectors governing NPC senescence and apoptosis remains unknown.

In this study, we identified ADM2 as a previously unrecognized downstream effector of the cGAS–STING pathway. Through integrated in vivo and in vitro analyses, we investigated whether D-allose attenuates NPC senescence and apoptosis by modulating the mtDNA–cGAS–STING–ADM2 axis. Our findings reveal that D-allose restores mitophagy, suppresses aberrant cGAS–STING activation, re-establishes ADM2 expression and ultimately alleviates IVDD. Together, this work provides a mechanistic framework and a translationally relevant strategy for rebalancing mitochondrial stress-driven innate immune signalling in IVDD.

## Materials and methods

### Ethics statement

The collection and use of human specimens were reviewed and approved by the Ethics Committee of the First Affiliated Hospital of Sun Yat-sen University. The animal experimental protocol was approved by the Institutional Animal Care and Use Committee at the First Affiliated Hospital of Sun Yat-sen University (No. [2024]160). All procedures complied with the ethical standards of the Declaration of Helsinki. Authorization from the Ministry of Science and Technology of China was obtained for the export of genetic materials. Written informed consent was obtained from all patients prior to inclusion. Tissue samples were collected during clinically required surgical procedures, and no financial compensation was offered to the patients involved.

### Human intervertebral disc with different grades of degeneration

From June to October 2025, we collected a total of 30 intervertebral disc (IVD) samples, including 14 from female and 16 from male patients, with a mean age of 47.81 ± 21.87 years. Disc degeneration was assessed using the Pfirrmann grading system. Normal IVD samples were sourced from patients with traumatic injuries, whereas degenerated IVDs were obtained from individuals diagnosed with degenerative spinal conditions, including disc herniation, spinal stenosis and degenerative scoliosis.

### Primary human nucleus pulposus cell culture

NPCs were cultured following the protocol described in our previous study^27^. Briefly, cells were maintained in Dulbecco’s Modified Eagle Medium (DMEM; Invitrogen, CA, USA) supplemented with 10% fetal bovine serum (FBS; Invitrogen) and antibiotics (Invitrogen), under standard conditions of 37 °C and 5% CO_2_ in a humidified incubator. Cells were harvested using a solution containing trypsin (0.25%) and EDTA (1 mM) (Invitrogen), and subsequently seeded onto 10-cm culture dishes.

NPCs were plated in six-well plates and cultured until approximately 80% confluence before experimental treatment. Cells were exposed to 500 µg/ml D-allose (MCE, HY-128741, USA; chemical structure shown in Supplementary Fig. 1a) for 24 h for subsequent analyses. To establish an inflammation-induced degenerative model, NPCs were incubated with 100 ng/ml recombinant human TNF (PeproTech, 300-01 A, USA) for 24 h. Cellular senescence was induced by treatment with 100 µM hydrogen peroxide (H_2_O_2_); cells were exposed to H_2_O_2_ for 6 h, followed by 24-h recovery in standard medium. This cycle was repeated three times to generate a stable senescent NPC phenotype. To modulate the cGAS–STING signalling pathway, NPCs were treated with the STING agonist diABZI (HY-112921A, MedChemExpress, USA) or the STING inhibitor H-151 (HY-112693, MedChemExpress, USA). For pathway inhibition, NPCs were incubated with 1 μM H-151 for 24 h, whereas activation was induced by treatment with 10 μM diABZI for 24 h.

### Mice model

The mouse model of IVDD was generated by annulus fibrosus puncture (AFP), following the procedure described in our previous work^7,27^. In this model, AFP was performed as a trans-annular puncture, in which a needle was inserted through the annulus fibrosus into the intervertebral disc to induce disc degeneration. C57BL/6 mice (8–9 weeks old, approximately 30 g; *n* = 5 per group) were housed under specific pathogen-free (SPF) conditions at a constant temperature of 23–25 °C and 50% relative humidity, with a 12-h light/dark cycle. During surgery, mice were placed in a prone position, and a midline incision was made along the dorsal surface. The left facet joint between the third and fourth lumbar vertebrae was carefully removed to expose the L3/4 intervertebral disc. A 26-gauge needle was then inserted to a depth of 1.0 mm into the disc parallel to the endplates for 30 s to induce disc degeneration. A clamp was applied as a depth limiter to avoid damage to adjacent structures. The muscle layer was closed with 3–0 silk sutures, and the skin was sutured using 4–0 nylon thread.

Mice in the D-allose + IVDD group were pretreated with intraperitoneal injections of D-allose (100 mg/kg/day) for 2 weeks before establishing an AFP-induced intervertebral disc degeneration (IVDD) model. After surgery, D-allose was administered intraperitoneally at the same dose for an additional 2 weeks.

### *Sting1* conditional knockout mice

*Sting1* conditional knockout (STING cKO) mice were generated using the Cre–LoxP recombination system by Cyagen (Suzhou, China). *Sting1*^flox/flox^ mice carrying LoxP sites flanking the *Sting1* gene were bred with *Col2a1*-Cre mice. The resulting *Sting1*^flox/+^; *Col2a1*-Cre mice were subsequently crossed with *Sting1*^flox/flox^ mice. Offspring with the genotype *Sting1*^flox/flox^; *Col2a1*-Cre were identified as STING cKO mice, whereas Cre-negative *Sting1*^flox/flox^ littermates served as controls. Genotypes were confirmed by PCR analysis of tail DNA (Supplementary material 1).

### Micro-CT and MRI

Eight weeks post-surgery, all mice underwent micro-CT and MRI assessments. Following anaesthesia, animals were positioned prone with the spine aligned in a natural posture. Micro-CT data were reconstructed and visualized using NRecon, DataViewer and CTvox software to generate three-dimensional images. The degree of disc degeneration was evaluated on T2-weighted MRI according to the Pfirrmann grading system. Briefly, the target disc was graded from I to V based on disc structure, T2 signal intensity, distinction between the nucleus pulposus and annulus fibrosus, and disc height. Pfirrmann grades were treated as ordinal variables and analysed using non-parametric statistical tests. Intervertebral disc height was measured on reconstructed micro-CT images as the disc space height between adjacent vertebral endplates at the target level, as described in our previous study^27^ and illustrated in Supplementary Fig. 3.

### RNA sequencing and analysis

The RNA sequencing was performed by Novogene (Beijing, China). The genes with *P* value < 0.05 and absolute |log2FoldChange | >1.0 in the DEG analysis were obtained for downstream analysis. Enrichment analysis was performed using the NovoMagic platform (Novogene, Beijing, China).

### Real-time reverse transcription quantitative PCR

Total RNA from NPC was extracted using an RNA-Quick Purification Kit (ES Science, China) following the manufacturer’s protocol. RNA concentration and purity were assessed with a NanoDrop spectrophotometer (Thermo Fisher Scientific, USA). The reverse transcription reaction was performed using PrimeScript RT Master Mix (Takara Bio, China). Real-time reverse transcription quantitative PCR (RT-qPCR) was performed with TB Green Premix Ex Taq II (Tli RNaseH Plus) (Takara Bio, China) on a QuantStudio 5 Real-Time PCR System (Thermo Fisher Scientific, USA). All primers were synthesized by Tianyi Huiyuan Biotech Co., Ltd., and primer sequences are provided in Supplementary Table 1.

### Cell transfection

ADM2 small interfering RNA (siRNA) and overexpression plasmids were generated by iGeneBio (Guangzhou, China). The sequences of the siRNA targets are provided in Supplementary Table 2. SiRNA negative control and the empty vector served as controls (CTR). Human NPCs were plated in six-well plates and cultured to 60–70% confluence. Reagents were diluted in Opti-MEM medium (Thermo Fisher, USA) according to the manufacturer’s instructions. Subsequently, the diluted siRNA, ADM2 overexpression plasmids, or empty vectors were separately mixed with diluted Lipofectamine 3000 (Invitrogen, USA) and incubated at room temperature for 10 min. All the lipid complexes were then separately added to cells for 48-h culture at 37 °C for subsequent assays.

### Western blot

Total proteins from NPCs were isolated, quantified and separated using 10% or 12.5% SDS-PAGE gels (Epizyme, China), followed by transfer onto 0.22-µm PVDF membranes (Millipore, USA). Membranes were blocked with 5% skimmed milk for 2 h at room temperature, then incubated overnight at 4 °C with primary antibodies, including anti-COL1A1 (1:1000, A1352, ABclonal), anti-COL2A1 (1:1000, A1560, ABclonal), anti-Aggrecan (1:1000, A8536, ABclonal), anti-p16 (1:1000, A11651, ABclonal), anti-p21 (1:1000, A19094, ABclonal), anti-γH2AX (1:1000, AP1555, ABclonal), anti-Cleaved-caspase3 (Cleaved-Cas3, 1:1000, A9664, ABclonal), anti-Bax (1:1000, A19684, ABclonal), anti-Bcl2 (1:1000, A19693, ABclonal), anti-PINK1 (1:1000, A25301, ABclonal), anti-Parkin (1:1000, A0968, ABclonal), anti-p62 (1:1000, A19700, ABclonal), anti-LC3A/B (1:1000, A27200PM, ABclonal), anti-cGAS (1:1000, A27527, ABclonal), anti-STING (1:1000, A21051, ABclonal), anti-Phospho-STING (1:1000, AP1369, ABclonal), anti-TBK1 (1:1000, A3458, ABclonal), anti-Phospho-TBK1 (1:1000, AP1026, ABclonal), anti-IRF3 (1:1000, A19717, ABclonal), anti-Phospho-IRF3 (1:1000, AP0995, ABclonal), anti-ADM2 (1:1000, PA5-72030, ThermoFisher), and HRP-conjugated anti-β-Tubulin (1:1000, AC030, ABclonal). After washing with TBS-T, the membranes were incubated with HRP-linked anti-rabbit IgG secondary antibody (1:10000, Cell Signaling Technology) for 2 h. The protein bands were detected with enhanced chemiluminescence detection reagents (Invitrogen).

### Enzyme-linked immunosorbent assay

Cell supernatants were collected after treating NP cells with H_2_O_2_ or D-allose. The IL-6 levels in supernatants were measured using an enzyme-linked immunosorbent assay (ELISA) kit (Elabscience, E-EL-H6156, China) according to the manufacturer’s protocol.

### Immunohistochemistry and histopathological analysis

Following deparaffinization, antigen retrieval and blocking with goat serum, human or mouse tissue sections were incubated overnight at 4 °C with primary antibodies, including anti-COL1A1 (1:200, A1352, ABclonal), anti-COL2A1 (1:300, A1560, ABclonal), anti-Aggrecan (1:200, A8536, ABclonal), anti-p21 (1:200, A19094, ABclonal) or anti-ADM2 (1:200, 23781-1-AP, Proteintech). Subsequently, sections were treated with the corresponding secondary antibody. Nuclear staining was performed using haematoxylin. Finally, the stained samples were examined, and images were acquired using a slide scanner (KFBIO, China).

### Immunofluorescence analysis

NPCs were fixed in 4% paraformaldehyde for 15 min and subsequently permeabilized with Triton X-100 for 10 min. Following washes with fresh PBS-T, the cells were blocked in 20% goat serum for 30 min. Cells were then incubated overnight at 4 °C with primary antibodies against p16 (1:200, AP11651, ABclonal), γH2AX (1:400, AP1555, ABclonal), and ds DNA Marker (1:200, sc-58749, Santa Cruz Biotechnology), followed by incubation with the corresponding secondary antibodies. Cell nuclei were counterstained with DAPI. Fluorescent images were acquired using an Olympus BX63 microscope (Olympus, Japan).

### SA-β-gal staining

SA-β-gal staining was performed using a Senescence β-Galactosidase Staining Kit (Beyotime, C0602, China) according to the manufacturer’s instructions. Representative images were acquired under bright-field illumination using an Olympus IX83 microscope (Olympus, Japan).

### Cytosolic mitochondrial DNA real-time quantitative PCR detection

Human primary NPCs were harvested using trypsin (0.25%) and EDTA (1 mM) (Invitrogen). The cell suspension was divided equally into two 1.50-ml microcentrifuge tubes. After centrifugation at 200 × *g* for 5 min, one pellet was frozen as the whole-cell control, whereas the other was resuspended in Buffer A (150 mM NaCl, 50 mM HEPES, 25 µg/ml Digitonin, pH 7.4) and incubated at room temperature for 10 min to selectively permeabilize the plasma membrane. The samples were then centrifuged at 1000 × *g* for 10 min at 4 °C to remove nuclei and intact cells, and the supernatant was further centrifuged at 17000 × *g* for 10 min to obtain the cytosolic fraction. DNA was then purified from this cytosolic fraction using the FastPure Cell/Tissue DNA Isolation Mini Kit (DC102-01, Vazyme, China).

The cytosolic fraction was subjected to qPCR analysis of mitochondrial gene MT-ND1 to quantify cytosolic mtDNA. The total DNA fraction was analysed for the nuclear gene 18S rDNA (encoding 18S rRNA), which served as an internal control for normalization between paired samples. qPCR was performed following the protocol described above. Primer sequences were provided in Supplementary Table 1.

### TUNEL assay

NPCs were washed with PBS and fixed in 4% paraformaldehyde for 10 min, followed by three additional washes with PBS. Apoptotic cells were then detected using a TUNEL detection kit (E-CK-A320, Elabscience) according to the manufacturer’s protocol.

### Flow cytometry

An Annexin V-FITC/PI Apoptosis Detection Kit (E-CK-A211, Elabscience) was applied to detect NPC apoptosis. Cells harvested from six-well plates were centrifuged at 300 × *g* for 5 min, then resuspended in 300 µl of 1× binding buffer. After 15–20 min of incubation with 5 µl of Annexin V-FITC and 5 µl of PI, 200 µl of 1× binding buffer was added, and the samples were immediately measured using a FACS Calibur flow cytometer (BD Bioscience, USA).

### Statistical analysis

All statistical analyses were performed using GraphPad Prism version 9.0. Data were first examined for normality using the Shapiro–Wilk test. For comparisons between the two groups, normally distributed data were analysed using an unpaired Student’s *t* test, whereas non-normally distributed or ordinal data (e.g. Pfirrmann grades and Histological score) were evaluated using the Mann–Whitney *U* test. Results are presented as mean ± standard deviation. A two-tailed *P* value < 0.05 was considered statistically significant.

## Results

### Intervertebral disc degeneration is a stress-driven disruption of nucleus pulposus cell homeostasis that remains therapeutically reversible by D-allose treatment

**Fig. 1:**
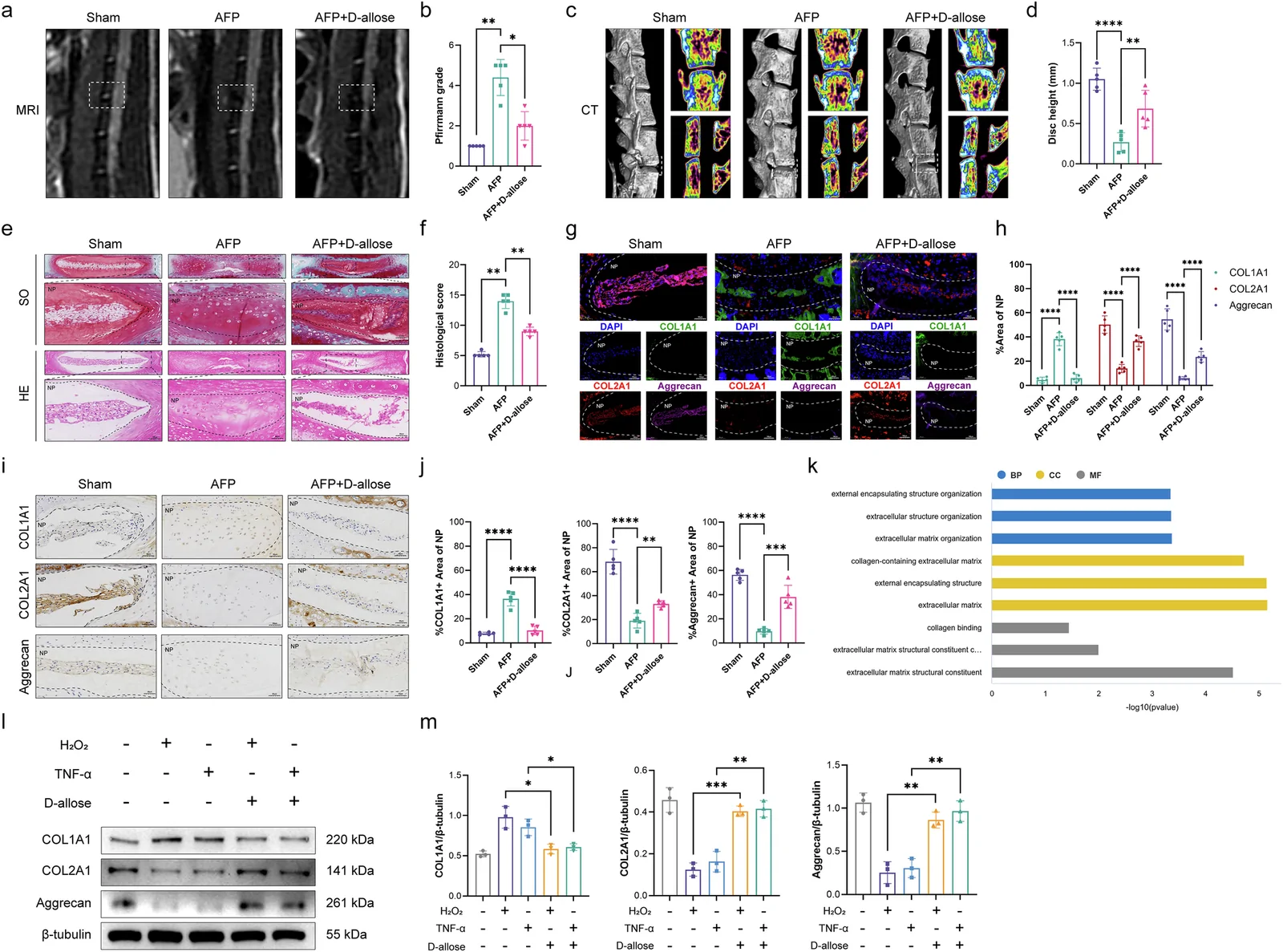
Intervertebral disc degeneration reflects a stress-associated disruption of nucleus pulposus cell homeostasis that remains therapeutically reversible. **a**,** b** T2-weighted MRI and corresponding Pfirrmann grading of lumbar intervertebral disc (IVDs) in mice from the sham, annulus fibrosus puncture (AFP) and AFP + D-allose groups. **c**,** d** Representative micro-CT images and disc height analysis of mice from sham, AFP and AFP + D-allose groups. **e**,** f** Haematoxylin–eosin (HE) and safranin O (SO) staining with histological assessment of mouse IVDs from the sham, AFP, and AFP + D-allose groups. **g**,** h** Immunofluorescence (IF) staining and quantitative analysis of COL1A1, COL2A1 and Aggrecan in mouse IVDs from the sham, AFP and AFP + D-allose groups. **i**,** j** Immunohistochemical (IHC) staining and quantitative analysis of COL1A1, COL2A1 and Aggrecan in mouse IVDs from the sham, AFP and AFP + D-allose groups. **k** Gene Ontology (GO) enrichment analysis of differentially expressed genes from RNA sequencing of NPC (intervertebral disc degeneration (IVDD) + D-allose vs IVDD). Significantly enriched extracellular matrix (ECM)-related terms are highlighted. **l**,** m** Western blot and quantitative analysis of COL1A1, COL2A1 and Aggrecan expression in NPCs treated with H_2_O_2_ or TNF, with or without D-allose. Data are presented as mean ± standard deviation. *P < 0.05; **P < 0.01; ***P < 0.001; ****P < 0.0001.

To determine whether IVDD represents a fixed structural collapse or a stress-associated but potentially reversible disruption of NPC homeostasis, we employed an annulus fibrosus puncture (AFP)-induced IVDD model and evaluated disc integrity using multimodal imaging and histological analyses. T2-weighted MRI revealed a marked loss of disc hydration and increased Pfirrmann grades in AFP-treated mice compared with sham controls (Fig. 1a, b), accompanied by significant disc height reduction on micro-CT analysis (Fig. 1c, d). Histological assessment using haematoxylin–eosin (HE) and safranin O (SO) staining further demonstrated pronounced disorganization of the NP structure (Fig. 1e, f).

Notably, systemic administration of D-allose significantly ameliorated these degenerative changes. D-allose-treated mice exhibited improved MRI signal intensity, lower Pfirrmann grades, preserved disc height and partial restoration of NP histoarchitecture (Fig. 1a–f).

Immunofluorescence staining revealed that AFP-induced degeneration was associated with reduced expression of the anabolic matrix components COL2A1 and aggrecan, together with aberrant accumulation of COL1A1 (Fig. 1g, h). D-allose treatment significantly reversed these matrix alterations in vivo, restoring COL2A1 and aggrecan expression while suppressing COL1A1 deposition (Fig. 1g, h). Consistently, immunohistochemical analysis further confirmed the corresponding changes in matrix protein distribution within NP tissues among the different groups (Fig. 1i, j).

To gain transcriptomic insight into these matrix alterations, Gene Ontology (GO) enrichment analysis of differentially expressed genes derived from RNA sequencing of NPC (IVDD + D-allose vs IVDD) was performed, highlighting significantly enriched ECM-related terms (Fig. 1k). In line with the histological and transcriptomic findings, Western blot analysis demonstrated corresponding changes in COL1A1, COL2A1 and Aggrecan protein levels in NPCs treated with H_2_O_2_ or TNF, with or without D-allose (Fig. 1l, m).

Collectively, these findings indicate that IVDD reflects a stress-associated disruption of NPC homeostasis that remains amenable to therapeutic rebalancing by D-allose.

### Oxidative stress drives premature senescence in nucleus pulposus cells, which is alleviated by D-allose-mediated stress-adaptive reprogramming

**Fig. 2:**
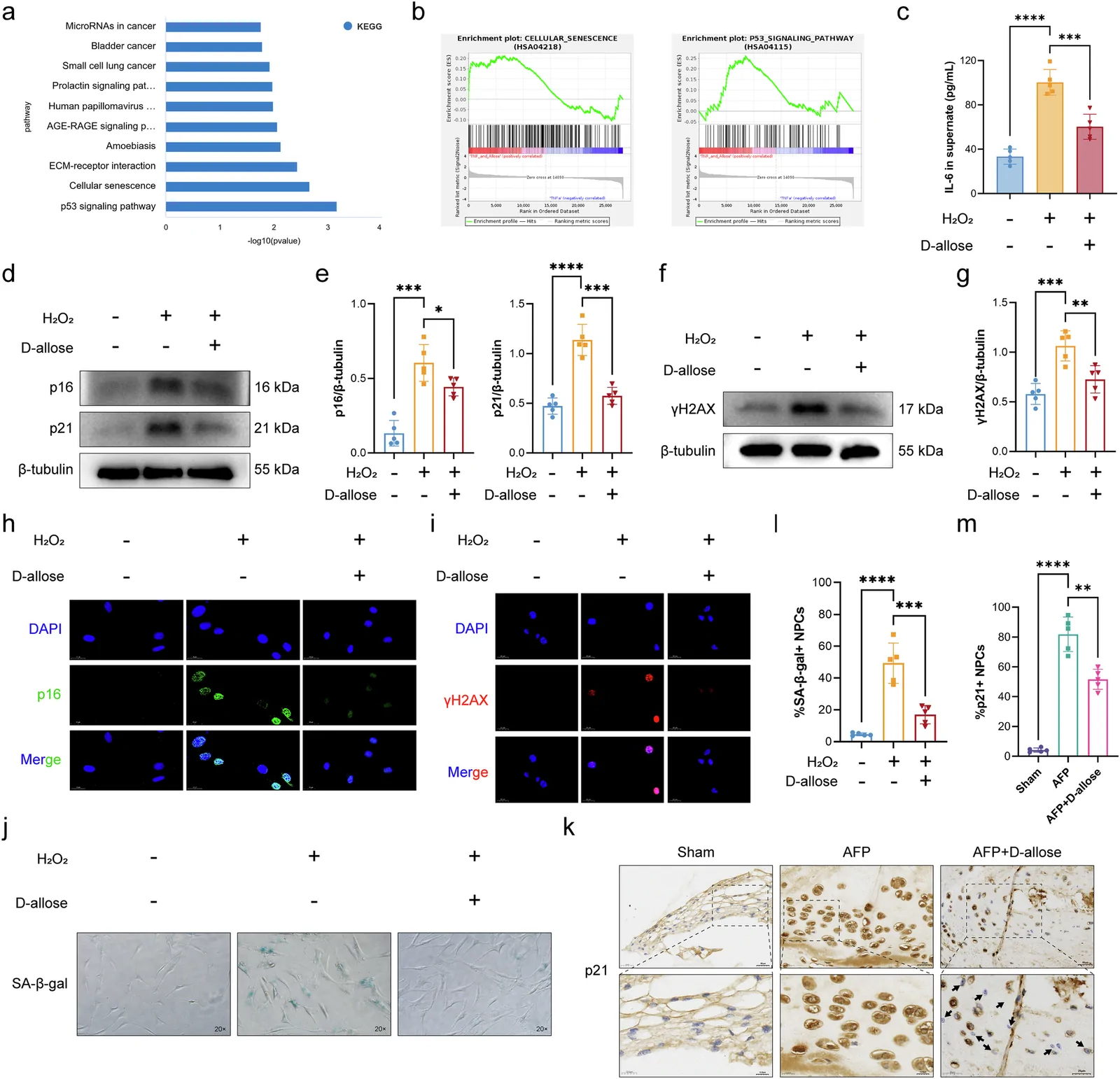
Oxidative stress induces nucleus pulposus cell senescence, which is attenuated by stress-adaptive reprogramming with D-allose. **a** Kyoto Encyclopedia of Genes and Genomes (KEGG) pathway enrichment of differentially expressed genes from RNA sequencing of nucleus pulposus cells (NPCs) (intervertebral disc degeneration (IVDD) + D-allose vs IVDD). Significantly enriched senescence-related terms are highlighted. **b** Gene Set Enrichment Analysis (GSEA) plots showing significant enrichment of the cellular senescence and p53 signalling pathways following D-allose treatment. **c** Enzyme-linked immunosorbent assay (ELISA) quantification of IL-6 secretion in NPCs treated with H_2_O_2_ with or without D-allose. **d**,** e** Western blot and quantification of senescence markers p16 and p21 expression. **f**, **g** Western blot and quantification of γH2AX levels. **h** Representative immunofluorescence (IF) staining of p16 in NPCs treated with H_2_O_2_ with or without D-allose. **i** Representative IF staining of γH2AX in NPCs treated with H_2_O_2_ with or without D-allose. **j**,** l** SA-β-gal staining images and quantification in NPCs treated with H_2_O_2_ with or without D-allose. **k**,** m** Immunohistochemistry (IHC) staining of p21 in mouse nucleus pulposus (NP) tissues from Sham, annulus fibrosus puncture (AFP) and AFP + D-allose groups.

To elucidate the cellular processes underlying the protective effects of D-allose, we performed transcriptomic profiling of stress-induced NPCs with or without D-allose treatment (Supplementary Fig. 1b). We identified 3399 (IVDD vs CTR), 1592 (D-allose vs CTR) and 568 DEGs (IVDD + D-allose vs IVDD) (Supplementary Fig. 1c–e). Kyoto Encyclopedia of Genes and Genomes (KEGG) enrichment analysis and Gene Set Enrichment Analysis (GSEA) revealed significant enrichment of senescence-associated pathways, including cellular senescence and p53 signalling, which were markedly attenuated upon D-allose exposure (Fig. 2a, b).

Consistent with these transcriptomic changes, oxidative stress induced by H_2_O_2_ resulted in robust secretion of the SASP IL-6 (Fig. 2c), upregulation of senescence markers p16 and p21 (Fig. 2d, e) and increased γH2AX expression (Fig. 2f, g). Immunofluorescence staining also confirmed the upregulation of p16 and γH2AX in stressed NPC (Fig. 2h, i). D-allose treatment significantly suppressed these senescence-associated features, as evidenced by reduced IL-6, p16, p21 and γH2AX expression (Fig. 2c–i). SA-β-gal staining further confirmed that D-allose attenuated stress-induced NPC senescence (Fig. 2j, l).

In vivo, immunohistochemical staining of NP tissues demonstrated increased p21 expression following AFP surgery, which was significantly reduced by D-allose administration (Fig. 2k, m). Collectively, these data indicate that D-allose effectively counteracts stress-induced NPC senescence at both transcriptional and functional levels.

### Inflammatory stress activates apoptotic programmes in nucleus pulposus cells that are restrained by D-allose-mediated cytoprotective modulation

**Fig. 3:**
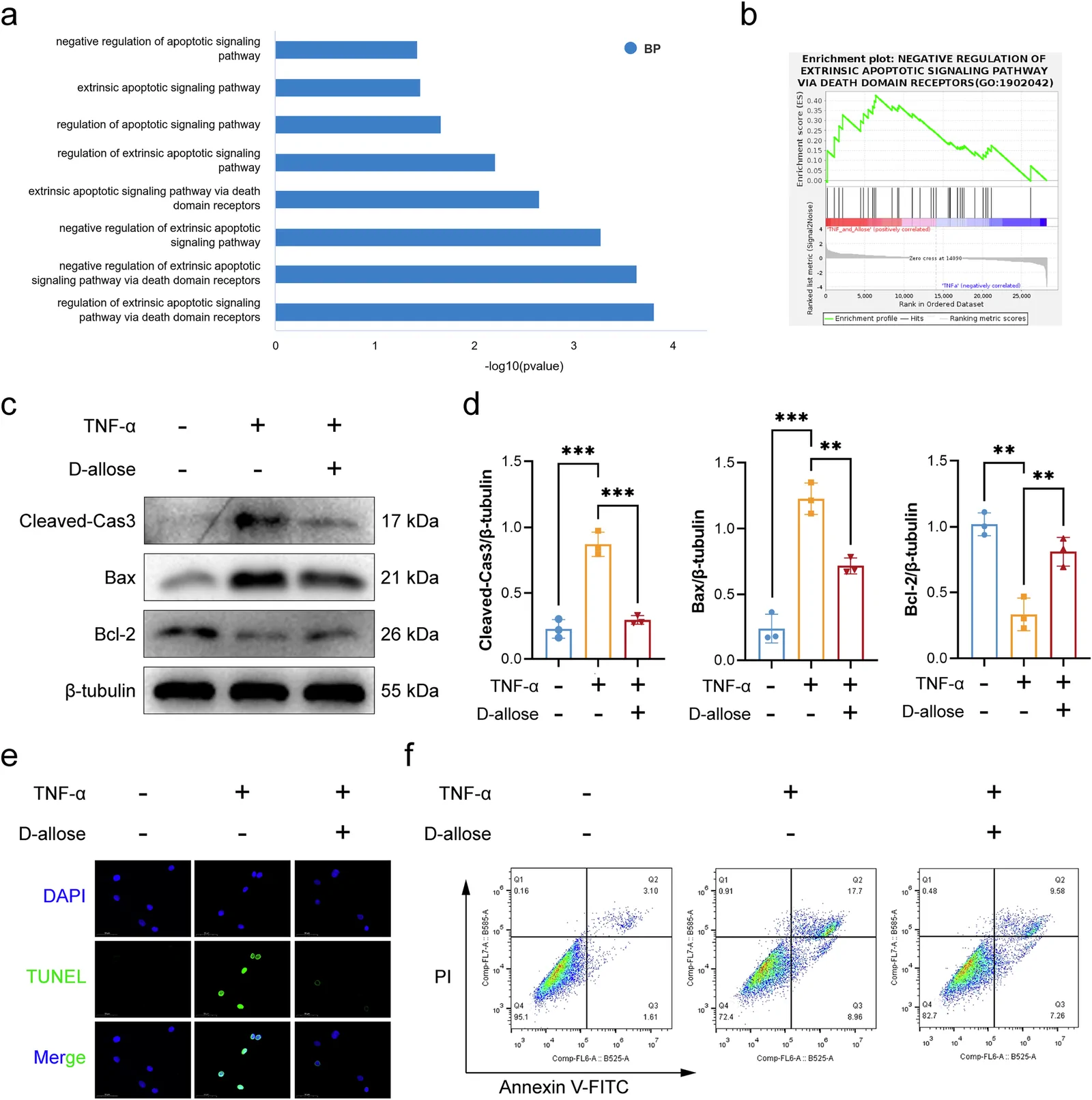
Inflammatory stress activates apoptotic signalling in nucleus pulposus cells, mitigated by D-allose-mediated cytoprotective modulation. **a** Gene Ontology (GO) enrichment analysis of biological processes associated with apoptosis-related pathways altered by D-allose treatment. **b** Gene Set Enrichment Analysis (GSEA) plots showing significant enrichment of the “negative regulation of extrinsic apoptotic signalling pathway via death domain receptors” in the D-allose-treated group. **c**,** d** Western blots and quantitative analysis of cleaved caspase-3 (Cleaved-Cas3), Bax and Bcl-2 expression in nucleus pulposus cells (NPCs) treated with TNF with or without D-allose. **e** TUNEL staining of NPCs showing apoptotic nuclei under different treatment conditions. **f** Flow cytometric analysis of Annexin V–FITC/PI staining demonstrating apoptotic cell populations. Data are presented as mean ± standard deviation. Statistical significance: *P < 0.05, **P < 0.01, ***P < 0.001.

Given the close interplay between senescence and apoptosis during IVDD, we next examined whether D-allose modulates apoptotic signalling in NPCs. Transcriptomic analyses revealed significant enrichment of the biological process associated with negative regulation of apoptotic signalling in stress-exposed NPCs treated with D-allose (Fig. 3a, b).

At the protein level, TNF stimulation markedly increased the expression of cleaved caspase-3 (Cleaved-Cas3) and Bax while reducing Bcl-2 expression, indicating activation of apoptotic cascades (Fig. 3c, d). D-allose treatment significantly reversed these changes, restoring the Bax/Bcl-2 balance and suppressing caspase-3 activation (Fig. 3c, d). TUNEL staining (Fig. 3e) and Annexin V–FITC/PI flow cytometric analysis (Fig. 3f) further confirmed that D-allose reduced apoptotic NPC populations under inflammatory stress conditions.

Together, these findings demonstrate that D-allose exerts a robust anti-apoptotic effect on NPCs, complementing its anti-senescent activity and contributing to the preservation of disc cellular viability.

### Degenerative stress-induced mitophagy dysfunction and cytosolic mitochondrial DNA accumulation in nucleus pulposus cells are reversed by D-allose

**Fig. 4:**
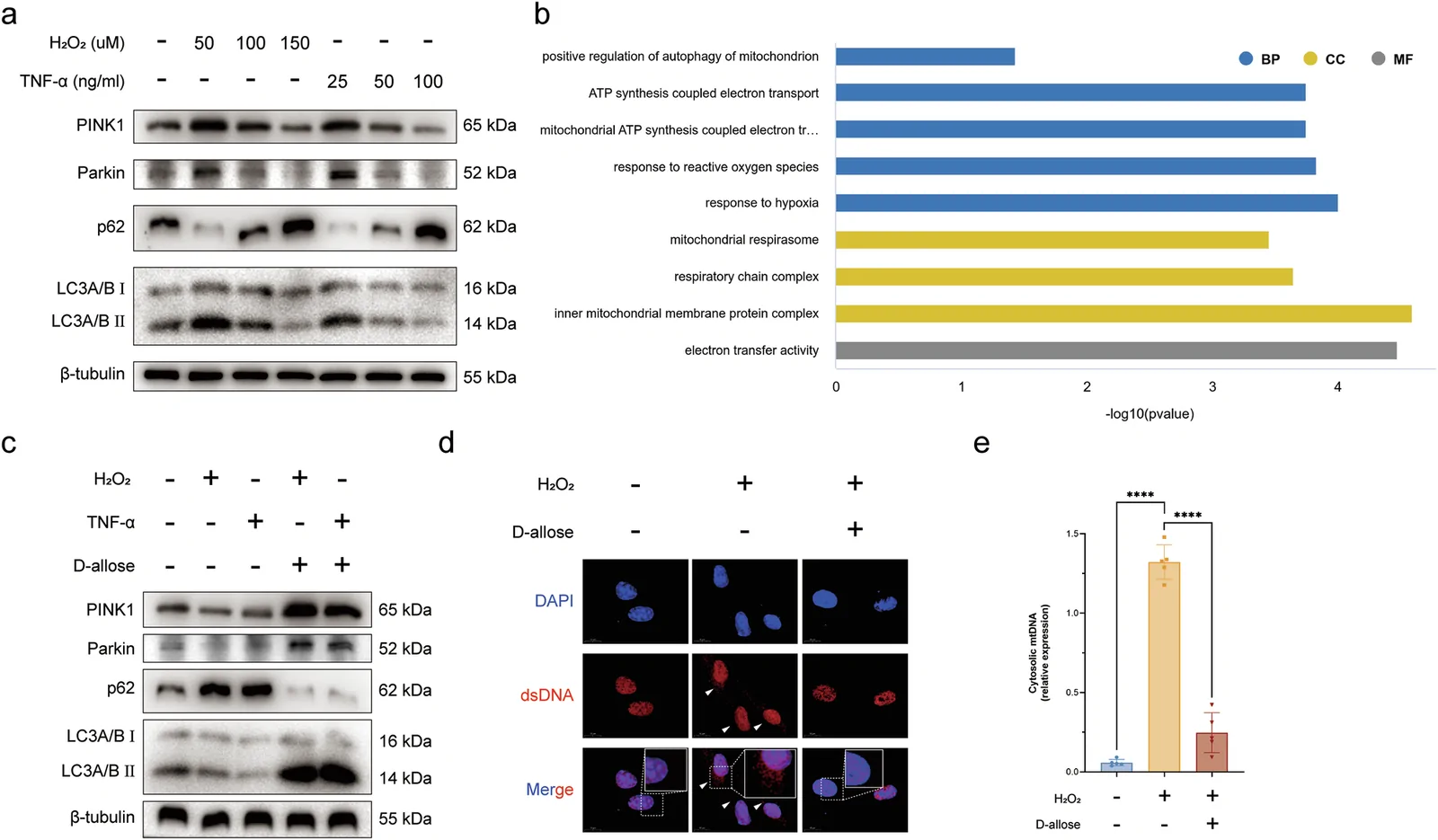
Degenerative stress impairs mitophagy and promotes cytosolic mitochondrial DNA accumulation, a process restrained by D-allose. **a** Western blot analysis of PINK1, Parkin, p62 and LC3 A/B expression in nucleus pulposus cells (NPCs) under different stimulations. **b** Gene Ontology (GO) enrichment analysis of biological processes, cellular component and molecular function associated with mitophagy-related pathways altered by D-allose treatment. **c** Western blot analysis of PINK1, Parkin, p62 and LC3 A/B expression in NPCs treated with H_2_O_2_ or TNF, with or without D-allose. **d** Representative immunofluorescence (IF) staining of dsDNA in NPCs treated with H_2_O_2_ with or without D-allose. **e** qPCR analysis of cytosolic mitochondrial DNA (mtDNA) in NPCs treated with H_2_O_2_ with or without D-allose. Data are presented as mean ± standard deviation. Statistical significance: *P < 0.05, **P < 0.01, ***P < 0.001.

Given the intimate links between cellular senescence, apoptosis and mitochondrial dysfunction, we investigated whether degenerative stressors impair mitochondrial quality control in NPCs and whether D-allose restores it. Under intense oxidative (H_2_O_2_) or inflammatory (TNF) stress, mitophagy was markedly impaired, as shown by decreased levels of PINK1 and Parkin, reduced LC3-II/LC3-I ratio and accumulation of p62 (Fig. 4a; Supplementary Fig. 2a). Gene Ontology enrichment analysis of D-allose-responsive transcripts further implicated mitochondrial organization and mitophagy pathways in stress alleviation (Fig. 4b). Consistent with these findings, D-allose treatment restored mitophagy-associated protein dynamics, reversing the stress-induced disruptions in both H_2_O_2_- and TNF-challenged NPCs (Fig. 4c; Supplementary Fig. 2b).

Immunofluorescence staining revealed pronounced accumulation of cytosolic double-stranded DNA in degenerated NPCs, which was significantly attenuated by D-allose (Fig. 4d). In agreement, qPCR analysis confirmed that D-allose markedly reduced cytosolic mtDNA levels (Fig. 4e).

Together, these data indicate that D-allose alleviates mitochondrial stress by restoring mitophagy, thereby limiting pathological mtDNA leakage into the cytoplasm.

### Stress-induced mitochondrial DNA–cGAS–STING signalling couples mitochondrial dysfunction to nucleus pulposus cell senescence, apoptosis and disc degeneration, and is therapeutically rebalanced by D-allose

**Fig. 5:**
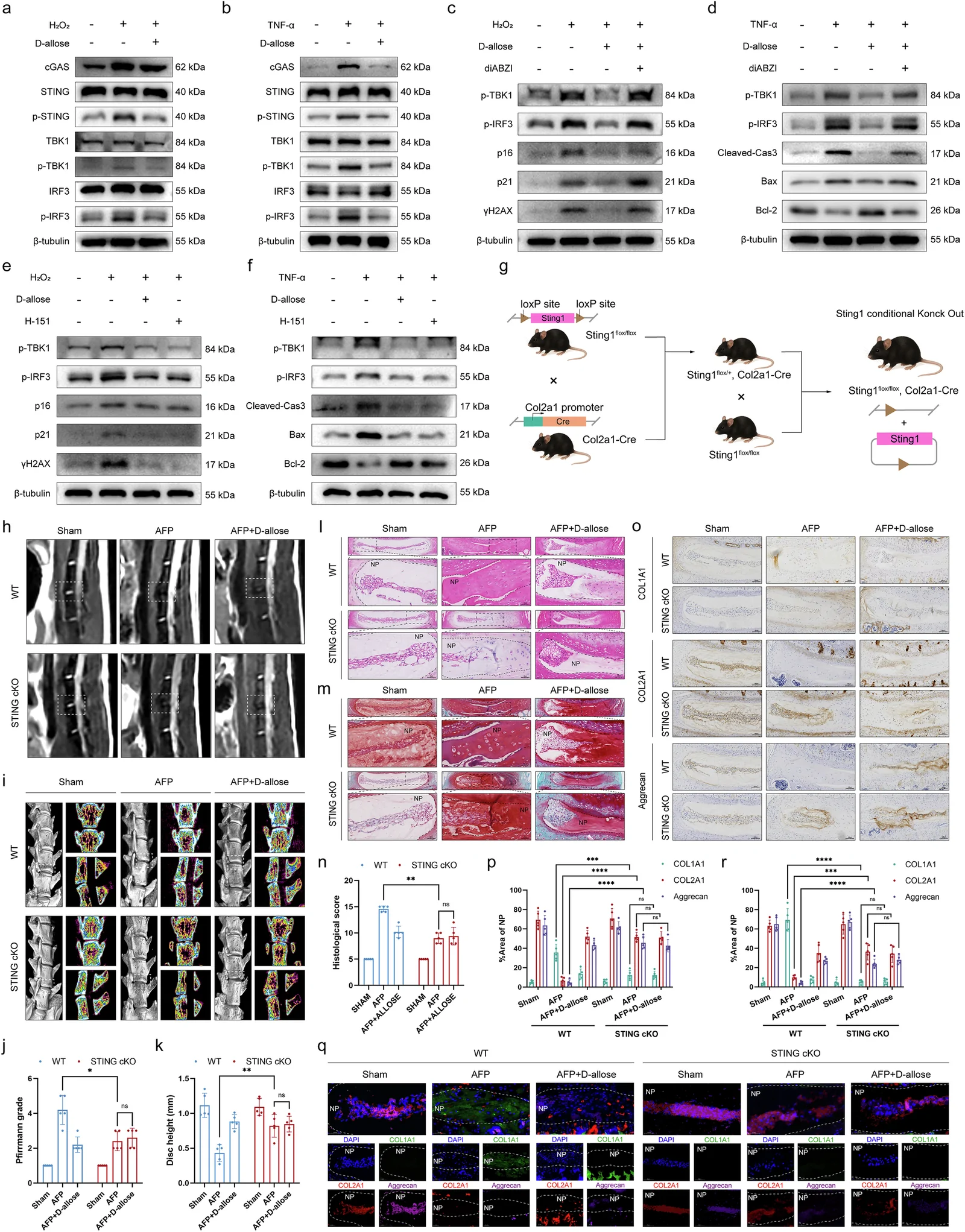
Stress-activated mitochondrial DNA–cGAS–STING signalling drives nucleus pulposus cell senescence and apoptosis to promote intervertebral disc degeneration and is therapeutically rebalanced by D-allose. **a**,** b** Western blot analysis of cGAS–STING signalling components and downstream TBK1 and IRF3 in nucleus pulposus cells (NPCs) stimulated with H_2_O_2_ or TNF, with or without D-allose treatment. **c**,** d** Western blot analysis of senescence- and apoptosis-related markers (p16, p21, γH2AX, cleaved caspase-3, Bax and Bcl-2), along with phosphorylated TBK1 and IRF3, in NPCs treated with H_2_O_2_ or TNF in the presence or absence of D-allose and the STING agonist diABZI. **e**,** f** Western blot analysis of the same senescence, apoptosis and cGAS–STING downstream markers in NPCs exposed to H_2_O_2_ or TNF, with or without D-allose and the STING inhibitor H-151. **g** Schematic illustration of the generation of nucleus pulposus (NP)-specific *Sting1* conditional knockout (cKO) mice. **h**,** j** T2-weighted MRI and corresponding Pfirrmann grading of lumbar intervertebral discs (IVDs) from wild type (WT) and STING cKO mice across the sham, annulus fibrosus puncture (AFP) and AFP + D-allose groups. **i**,** k** Representative micro-CT images and disc height analysis of WT and STING cKO mice from the sham, AFP and AFP + D-allose groups. **l**–**n** Haematoxylin–eosin (HE) and safranin O (SO) staining with histological scoring of IVDs from WT and STING cKO mice in the sham, AFP and AFP + D-allose groups. **o**,** p** Immunohistochemical (IHC) staining and quantitative analysis of COL1A1, COL2A1 and Aggrecan in WT and STING cKO mouse IVDs from the sham, AFP and AFP + D-allose groups. **q**,** r** Immunofluorescence (IF) staining and quantification of COL1A1, COL2A1 and Aggrecan in WT and STING cKO mouse IVDs from the sham, AFP and AFP + D-allose groups. Data are presented as mean ± standard deviation. Statistical significance: *P < 0.05, **P < 0.01, ***P < 0.001.

Because cytosolic mtDNA represents a major danger signal linking mitochondrial stress to innate immune activation, we next examined whether the mtDNA-driven cGAS–STING pathway is engaged under degenerative conditions. Stress stimulation robustly activated the cGAS–STING pathway in NPCs, as evidenced by increased cGAS expression, enhanced phosphorylation of STING and activation of downstream TBK1 and IRF3, reflected by elevated levels of phosphorylated TBK1 (p-TBK1) and phosphorylated IRF3 (p-IRF3) (Fig. 5a, b; Supplementary Fig. 2c, d). Notably, D-allose treatment markedly suppressed this aberrant pathway activation under both oxidative and inflammatory stress conditions (Fig. 5a, b; Supplementary Fig. 2c, d).

Pharmacological modulation of STING further confirmed the functional relevance of this pathway. Activation of STING using diABZI abolished the protective effects of D-allose on senescence- and apoptosis-related markers (Fig. 5c, d; Supplementary Fig. 2e, f), whereas inhibition of STING with H-151 phenocopied the effects of D-allose (Fig. 5e, f; Supplementary Fig. 2g, h). Together, these results support cGAS–STING suppression as an important contributor to D-allose-mediated NPC protection.

To further validate this mechanism in vivo, we generated nucleus pulposus-specific *Sting1* conditional knockout (*Sting1*^flox/flox^*Col2a1*-Cre, STING cKO) mice using the Cre–LoxP recombination system (Fig. 5g) and subjected them to AFP-induced IVDD. Compared with wild type controls, STING cKO mice exhibited marked resistance to disc degeneration, as reflected by lower Pfirrmann grades (Fig. 5h, j), maintenance of disc height (Fig. 5i, k), improved histological architecture (Fig. 5l-n) and restored ECM components (Fig. 5o–r). Notably, administration of D-allose failed to confer additional protective effects in STING-deficient discs (Fig. 5h–r), indicating that D-allose mitigates NPC senescence and apoptosis predominantly through suppression of the cGAS–STING pathway.

Collectively, these experiments establish the mtDNA-cGAS–STING axis as a critical pathogenic mediator of stress-induced NPC senescence and apoptosis and identify this axis as the principal molecular target through which D-allose exerts its protective effects in IVDD.

### ADM2 is identified as a stress-responsive downstream effector of the mitochondrial DNA–cGAS–STING axis during intervertebral disc degeneration

Having established cGAS–STING signalling as a critical mediator linking mitochondrial stress to NPC senescence and apoptosis, we next sought to identify endogenous downstream effectors that translate this innate immune activation into cell fate decisions.

**Fig. 6:**
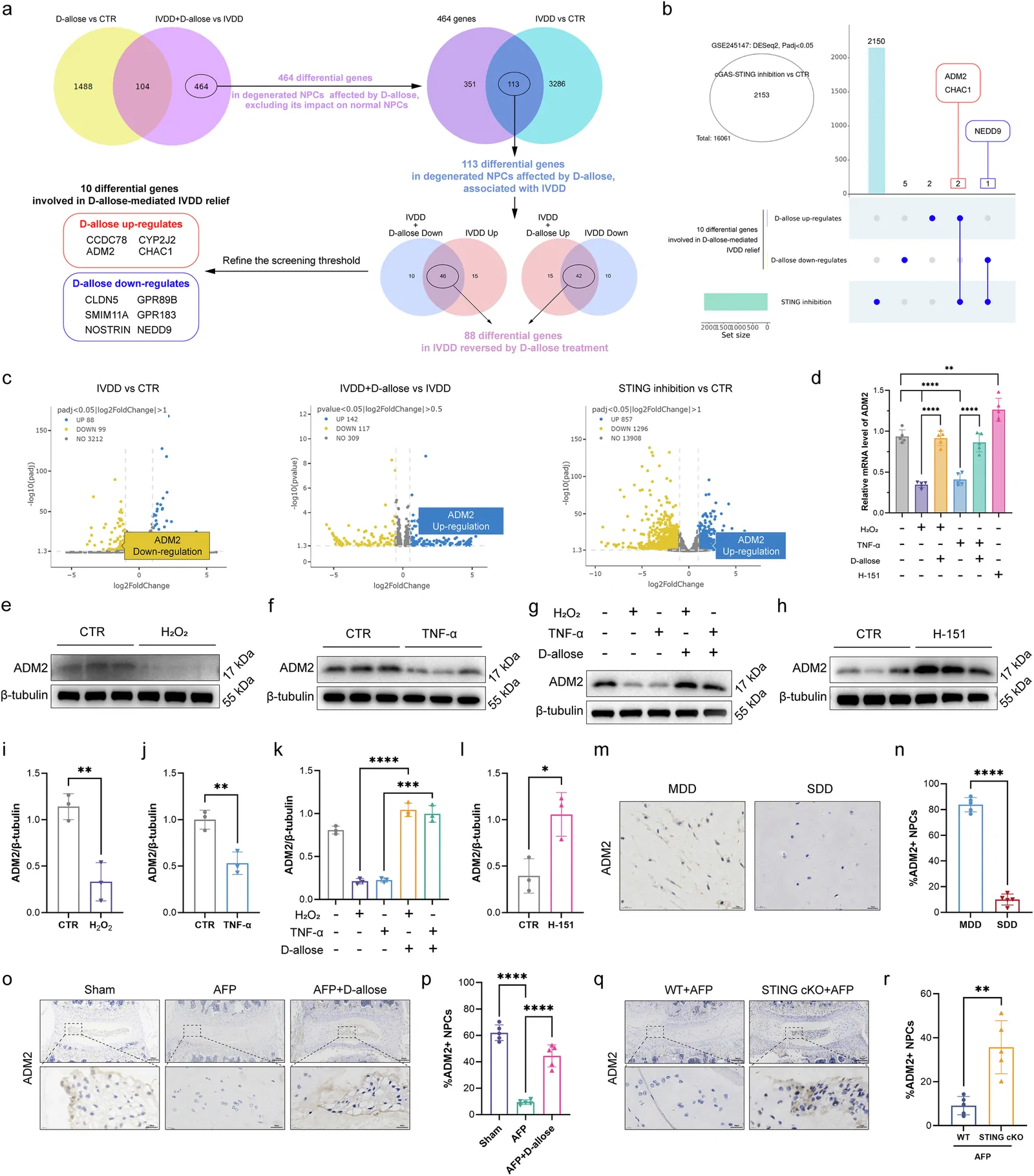
ADM2 functions as a stress-responsive downstream effector of the mitochondrial DNA–cGAS–STING axis in intervertebral disc degeneration. **a** Identification process of 10 differential genes involved in D-allose-mediated intervertebral disc degeneration (IVDD) relief. **b** Upset plot screening of genes regulated by the cGAS–STING pathway and potentially involved in D-allose-mediated attenuation of IVDD. **c** Volcano plots of ADM2 expression in different gene sets. **d** Real-time quantitative PCR (RT-qPCR) analysis of ADM2 expression in nucleus pulposus cells (NPCs) treated with H_2_O_2_ or TNF, with or without D-allose, and with STING inhibitor H-151. **e**,** i** Western blot and quantitative analysis of ADM2 expression in NPCs treated with H_2_O_2_. **f**,** j** Western blot and quantitative analysis of ADM2 expression in NPCs treated with TNF. **g**,** k** Western blot and quantitative analysis of ADM2 expression in NPCs treated with H_2_O_2_ or TNF, with or without D-allose. **h**,** l** Western blot and quantitative analysis of ADM2 expression in NPCs treated with H-151. **m**,** n** Immunohistochemistry (IHC) staining and quantitative analysis of ADM2 in human mild degenerated disc (MDD) and severe degenerated disc (SDD) nucleus pulposus (NP) tissues. **o**,** p** IHC staining and quantitative analysis of ADM2 in mouse NP tissues from Sham, annulus fibrosus puncture (AFP) and AFP + D-allose groups. **q**,** r** IHC staining and quantitative analysis of ADM2 in NP tissues from wild type (WT) and *Sting1* conditional knockout (STING cKO) mice after AFP surgery.

Differentially expressed genes regulated by D-allose under degenerative stress were first filtered to exclude transcripts altered under basal conditions, thereby enriching for stress-responsive targets relevant to IVDD. Intersection of these genes with IVDD-associated expression signatures yielded a focused set of ten candidate genes potentially involved in D-allose–mediated disc protection (Fig. 6a). To further prioritize candidates regulated by cGAS–STING signalling, we analysed an independent RNA-seq dataset of NPCs treated with the STING inhibitor H-151 (GSE245147)^28^. Cross-comparison revealed three overlapping genes — *ADM2*, *CHAC1* and *NEDD9* — that were consistently regulated under conditions of cGAS–STING attenuation (Fig. 6b). Among these candidates, ADM2 exhibited a consistent direction of regulation across the relevant comparisons: it was decreased under IVDD-associated degenerative conditions, restored by D-allose treatment and similarly increased after STING inhibition (Fig. 6c; Supplementary Table 3). Given its reported cytoprotective roles in cellular stress responses, inflammation regulation and cell survival, this expression pattern supported ADM2 as a biologically relevant downstream candidate for further functional validation.

At the expression level, ADM2 was markedly suppressed in NPCs exposed to oxidative or inflammatory stress, consistent with activation of mtDNA-driven innate immune signalling (Fig. 6d–f, i, j). Notably, restoration of mitochondrial homeostasis by D-allose treatment robustly rescued ADM2 expression at both mRNA and protein levels (Fig. 6d, g, k). A comparable recovery of ADM2 was observed upon pharmacological inhibition of STING, indicating that ADM2 suppression is tightly coupled to cGAS–STING pathway activation (Fig. 6d, h, l).

To assess the in vivo relevance of ADM2 dysregulation during IVDD, we first examined human NP tissues. Immunohistochemical analysis revealed a progressive loss of ADM2 expression in severely degenerated human discs compared with mildly degenerated controls (Fig. 6m, n), indicating that ADM2 deficiency is associated with advanced IVDD pathology. Consistently, in the AFP-induced mouse model of IVDD, ADM2 expression in NP tissue was markedly reduced following disc injury (Fig. 6o, p). Notably, systemic administration of D-allose significantly preserved ADM2 expression in degenerating discs (Fig. 6o, p), supporting a stress-responsive and therapeutically reversible regulation of ADM2 in vivo.

To further determine whether ADM2 expression is regulated by cGAS–STING signalling, we next examined NP-specific *Sting1* conditional knockout mice subjected to AFP-induced IVDD. Knockout of STING in NP tissue markedly attenuated the AFP-induced loss of ADM2 expression, maintaining higher ADM2 levels compared with wild type controls (Fig. 6q, r). These findings indicate that STING activity is required for stress-associated suppression of ADM2 in degenerating discs.

Collectively, these observations position ADM2 as a stress-responsive effector downstream of cGAS–STING signalling in IVDD, and implicate its preservation by D-allose as a key component of the therapeutic response.

### ADM2 governs nucleus pulposus cell fate and is required for resistance to senescence and apoptosis

**Fig. 7:**
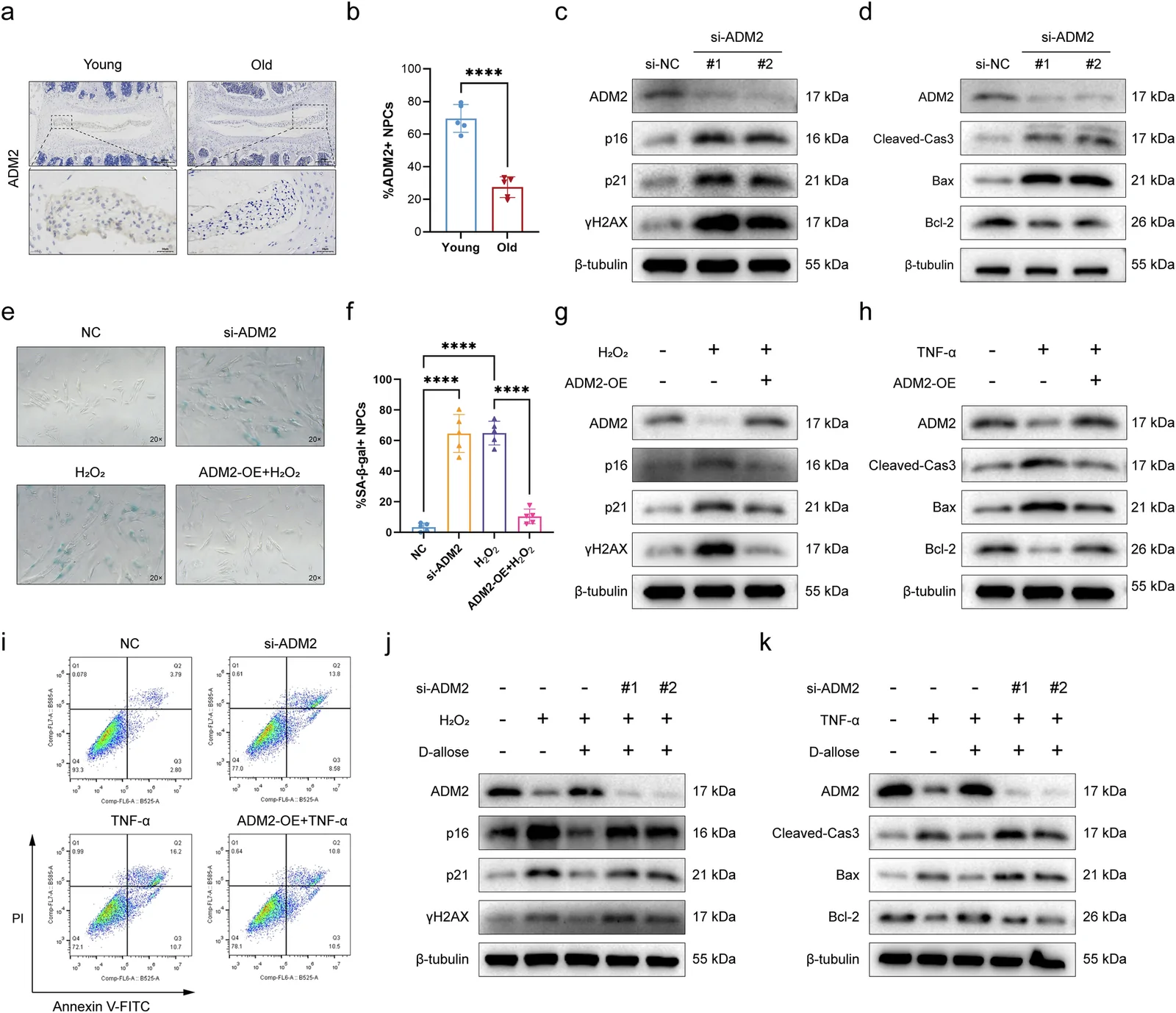
ADM2 governs nucleus pulposus cell fate and is required for resistance to senescence and apoptosis. **a**,** b** Immunohistochemistry (IHC) staining and quantitative analysis of ADM2 expression in nucleus pulposus (NP) tissue from mice of different ages. **c**,** d** Western blot analysis of ADM2 and senescence- and apoptosis-related markers expression following ADM2 knockdown. **e**,** f** SA-β-gal staining images and quantification in ADM2 knockdown nucleus pulposus cells (NPCs) and NPCs treated with H_2_O_2_, with or without ADM2 overexpression. **g**,** h** Western blot analysis of ADM2 and senescence- and apoptosis-related markers expression following ADM2 overexpression. **i** Flow cytometric analysis of Annexin V–FITC/PI staining in ADM2 knockdown NPCs and NPCs treated with H_2_O_2_, with or without ADM2 overexpression. **j**,** k** Western blot analysis of ADM2 and senescence- and apoptosis-related markers expression in the presence or absence of ADM2 knockdown or D-allose treatment.

To define the functional significance of ADM2 in NPC fate regulation, we next performed loss- and gain-of-function analyses. ADM2 expression declined progressively with ageing in murine NP tissues, suggesting a potential association between ADM2 insufficiency and age-related IVDD (Fig. 7a, b). In vitro, genetic knockdown of ADM2 induced pronounced cellular dysfunction in NPCs. Specifically, ADM2 silencing led to robust upregulation of senescence markers p16, p21 and γH2AX (Fig. 7c, Supplementary Fig. 2i), accompanied by increased expression of pro-apoptotic proteins and concomitant downregulation of the anti-apoptotic factor Bcl-2 (Fig. 7d, Supplementary Fig. 2j). Functionally, ADM2 knockdown significantly increased the proportion of SA-β-gal-positive NPCs (Fig. 7e, f) and markedly elevated apoptotic NPC populations as assessed by Annexin V–FITC/PI flow cytometry (Fig. 7i), demonstrating that loss of ADM2 directly drives NPC senescence and apoptosis.

Conversely, enforced ADM2 overexpression exerted a pronounced cytoprotective effect. ADM2 overexpression suppressed senescence-associated (Fig. 7g, Supplementary Fig. 2k) and apoptosis-associated (Fig. 7h, Supplementary Fig. 2l) protein changes, reduced SA-β-gal positivity (Fig. 7e, f), and significantly decreased apoptotic rates (Fig. 7i), under degenerative stress conditions.

Importantly, functional interference with ADM2 profoundly affected the therapeutic effect of D-allose. Western blot analyses revealed that ADM2 knockdown substantially abolished the ability of D-allose to suppress senescence markers (Fig. 7j, Supplementary Fig. 2m) and to rebalance pro- and anti-apoptotic signalling (Fig. 7k, Supplementary Fig. 2n) in stressed NPCs.

Collectively, these data establish ADM2 as a critical downstream effector required for D-allose-mediated cytoprotection, functionally linking suppression of the mtDNA–cGAS–STING pathway to restoration of NPC homeostasis and the attenuation of IVDD.

## Discussion

**Fig. 8:**
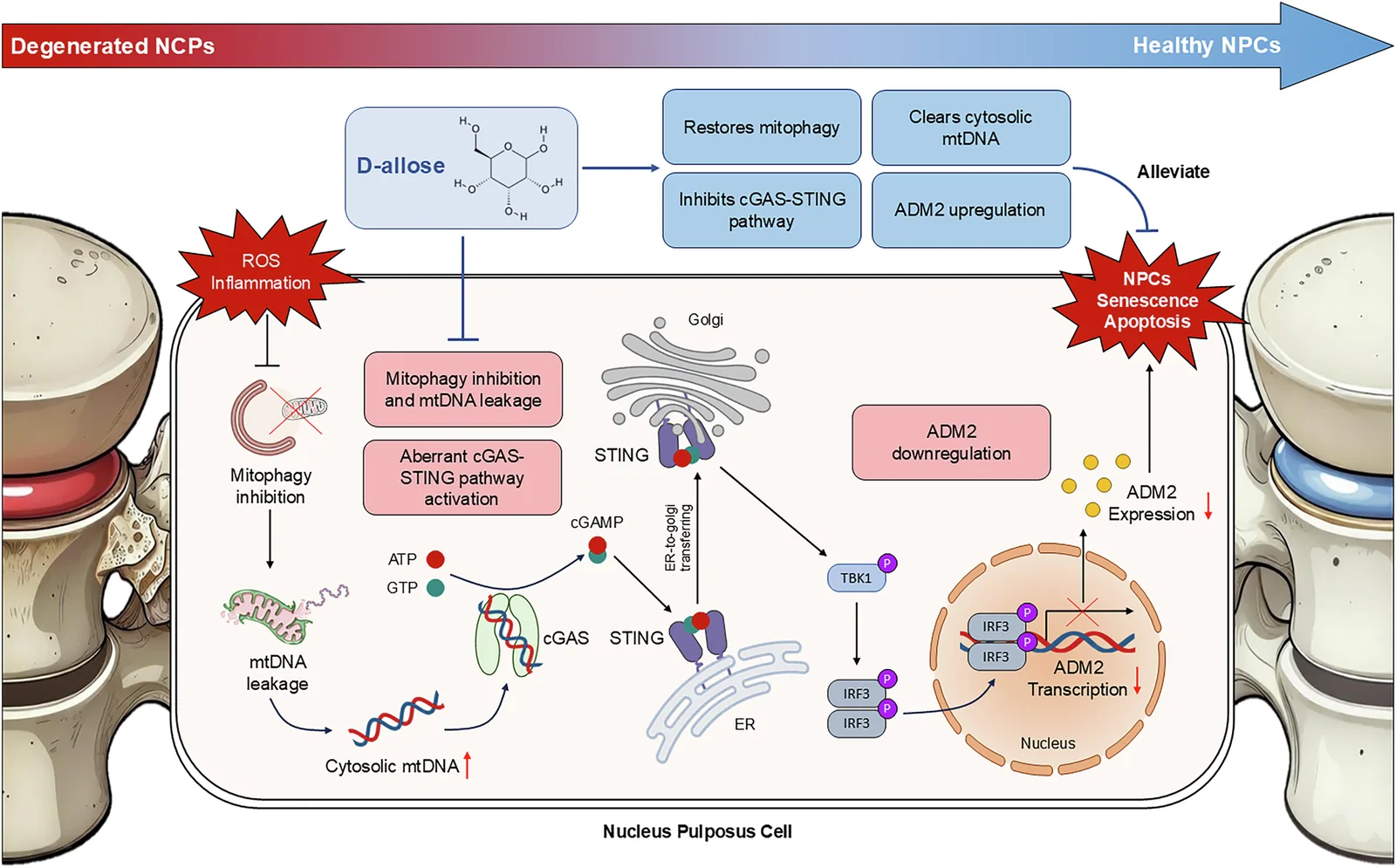
D-allose rebalances stress-driven mitochondrial DNA (mtDNA)–cGAS–STING–ADM2 signaling to protect against intervertebral disc degeneration. NPCs, nucleus pulposus cells; ROS, reactive oxygen species.

IVDD is increasingly recognized as a long-term stress response in which NPCs gradually lose their ability to maintain the ECM and survive. In this study, we connect mitochondrial dysfunction to NPC fate and IVDD through a clear chain of events. We show that impaired mitophagy promotes cytosolic mtDNA accumulation, leading to aberrant activation of the cGAS–STING pathway, suppression of the cytoprotective effector ADM2 and subsequent induction of NPC senescence and apoptosis. Importantly, we identify D-allose as a naturally occurring intervention capable of rebalancing this mtDNA–cGAS–STING–ADM2 axis and restoring disc cellular homeostasis (Fig. 8).

Accumulating evidence has implicated mitophagy dysfunction, and innate immune activation in IVDD^16,29^. However, these processes have largely been studied in isolation. Here, we integrate these observations into a unified pathogenic framework in which impaired mitochondrial quality control acts upstream to trigger mtDNA-driven innate immune signalling. Cytosolic mtDNA serves as a key danger signal that activates cGAS–STING signalling in NPCs, thereby promoting degenerative cell fate programmes. Genetic ablation of STING in NP tissue confers marked resistance to disc degeneration in vivo, underscoring the functional importance of this pathway in linking mitochondrial stress to tissue-level degeneration.

A key advance of this work is the identification of ADM2 as an endogenous downstream effector that translates cGAS–STING activation into NPC fate decisions. Through integrative transcriptomic screening across degenerative, STING-inhibited and D-allose-treated conditions, ADM2 emerged as a convergent target of stress-responsive innate immune signalling. ADM2 expression is progressively lost in human and murine degenerated discs, restored upon suppression of cGAS–STING signalling, and functionally required to maintain NPC resistance to senescence and apoptosis. Although other candidate genes were identified in the screening process, ADM2 displayed the most robust stress responsiveness, consistent directional regulation across datasets, and direct functional relevance to NPC survival^30–32^. These findings position ADM2 as a previously unrecognized output node of the mtDNA–cGAS–STING axis and suggest that degeneration-associated innate immune activation compromises disc integrity not only through inflammation, but also through silencing of intrinsic cytoprotective programmes.

Our study further identifies D-allose as a previously unrecognized modulator of this pathogenic axis. Rather than acting as a conventional pathway inhibitor, D-allose re-establishes cellular homeostasis by coordinately restoring mitochondrial quality control, limiting mtDNA-driven innate immune activation, and preserving ADM2-dependent cytoprotective signalling. This integrative mode of action highlights a distinct regulatory capacity of D-allose in reprogramming stress-induced NPC fate towards homeostasis.

Importantly, unlike conventional pharmacological agents that are synthetic or associated with adverse effects that limit their long-term applicability^33,34^, D-allose possesses properties that are particularly advantageous for chronic degenerative diseases such as IVDD. As a naturally occurring rare sugar with ultra-low caloric content and negligible toxicity, D-allose is very suitable for long-term administration^24,35,36^. Its dual identity as both a bioactive stress-modulating molecule and a potential sugar substitute further distinguishes it from traditional small-molecule drugs, offering the possibility of sustained therapeutic benefit with minimal metabolic or systemic burden^37^. These features are especially relevant in ageing populations, where cumulative drug toxicity and metabolic load represent major clinical challenges.

Although ADM2 was identified as a key downstream effector of cGAS–STING signalling in NPCs, the molecular mechanisms governing its regulation remain to be defined. In addition, although our data support a role for restored mitophagy in reducing cytosolic mtDNA accumulation after D-allose treatment, mitophagy-independent mechanisms may also contribute to this effect and warrant further investigation. From a translational perspective, the pharmacokinetic profile of D-allose during IVDD treatment was not examined in this study. Given the relatively avascular nature of the intervertebral disc, future studies should determine the systemic pharmacokinetics of D-allose, as well as its time-dependent distribution and retention within disc tissue. In addition, the routes by which D-allose reaches the IVD, together with the optimal route of administration, dosing regimen, treatment window and long-term safety profile, require further investigation. Addressing these issues will be essential for accelerating the clinical translation of D-allose and for assessing whether its protective effects can be extended to human disc tissue or other degenerative conditions.

In summary, our findings uncover a mechanistic axis in which mitophagy dysfunction, mtDNA-driven cGAS–STING signalling and ADM2-dependent cytoprotection converge to govern NPC fate. By demonstrating that this axis is both mechanistically tractable and therapeutically reversible using a safe, naturally derived molecule, this work provides a conceptual and translational framework for targeting stress-induced degeneration in IVDD and potentially other ageing-related diseases.

## Supplementary information

Supplementary Information

## Data Availability

The datasets used and analysed during the current study are available from the corresponding author on reasonable request.
