# Supplementary material for: D-allose reprogrammes the mitophagy–mtDNA–cGAS–STING–ADM2 axis to restore nucleus pulposus cell homeostasis in intervertebral disc degeneration
Source: Exp Mol Med. 2026 Oct 1;58(10):3357–69. doi: 10.1038/s12276-026-01844-7 (PMC13737178; doi:10.1038/s12276-026-01844-7)
Supplement: Supplementary file 1 [file 12276_2026_1844_MOESM1_ESM.pdf]

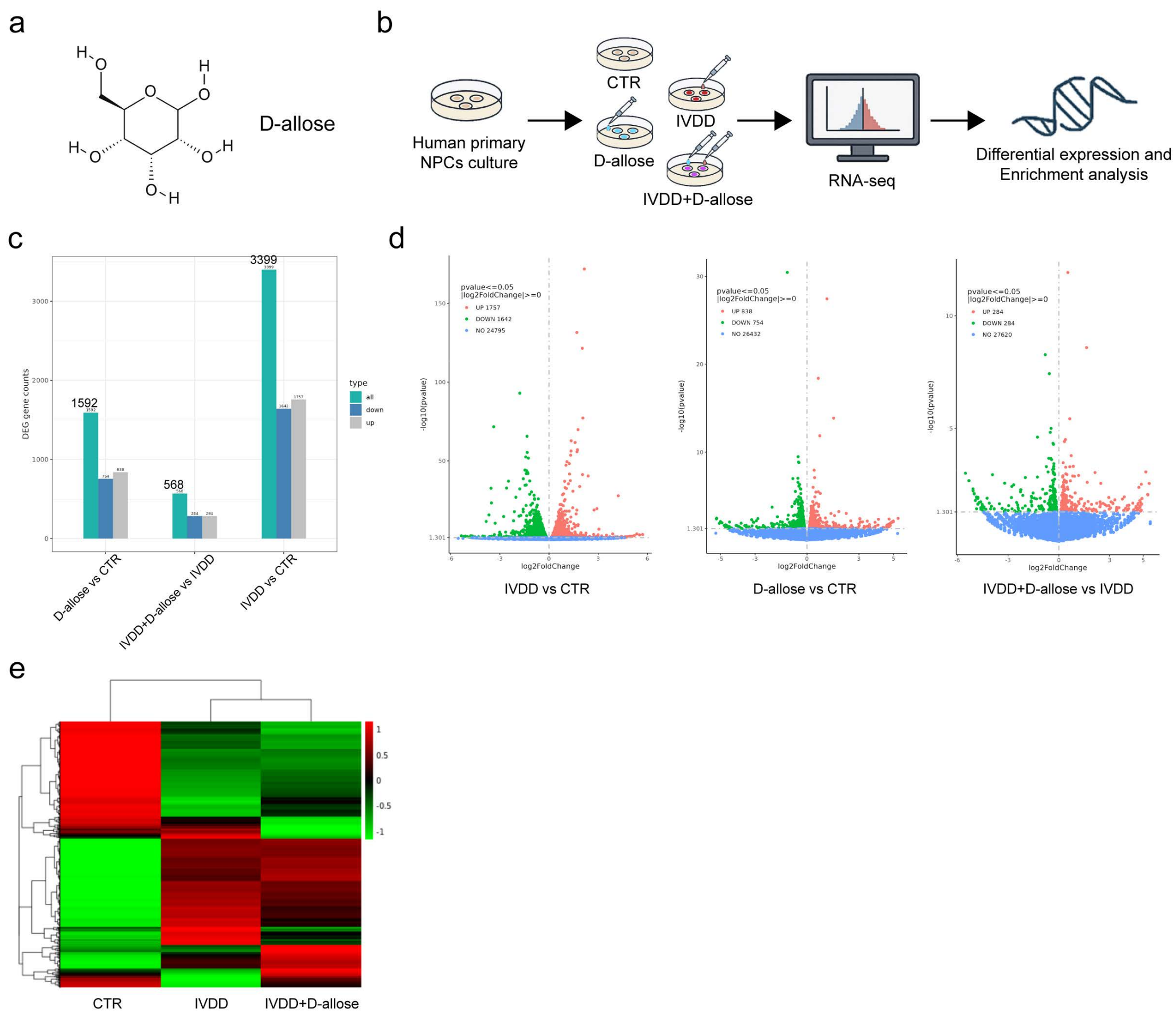

Supplementary Fig. 1 Transcriptomic profiling of D-allose-treated human nucleus pulposus cells.

(a) Chemical structure of D-allose.

(b) Schematic illustration of the RNA sequencing (RNA-seq) workflow.

(c) Numbers of differentially expressed genes (DEGs) in the indicated comparisons.

(d) Volcano plots showing DEGs in the indicated comparisons.

(e) Hierarchical clustering heatmap showing the expression patterns of DEGs among the indicated groups.

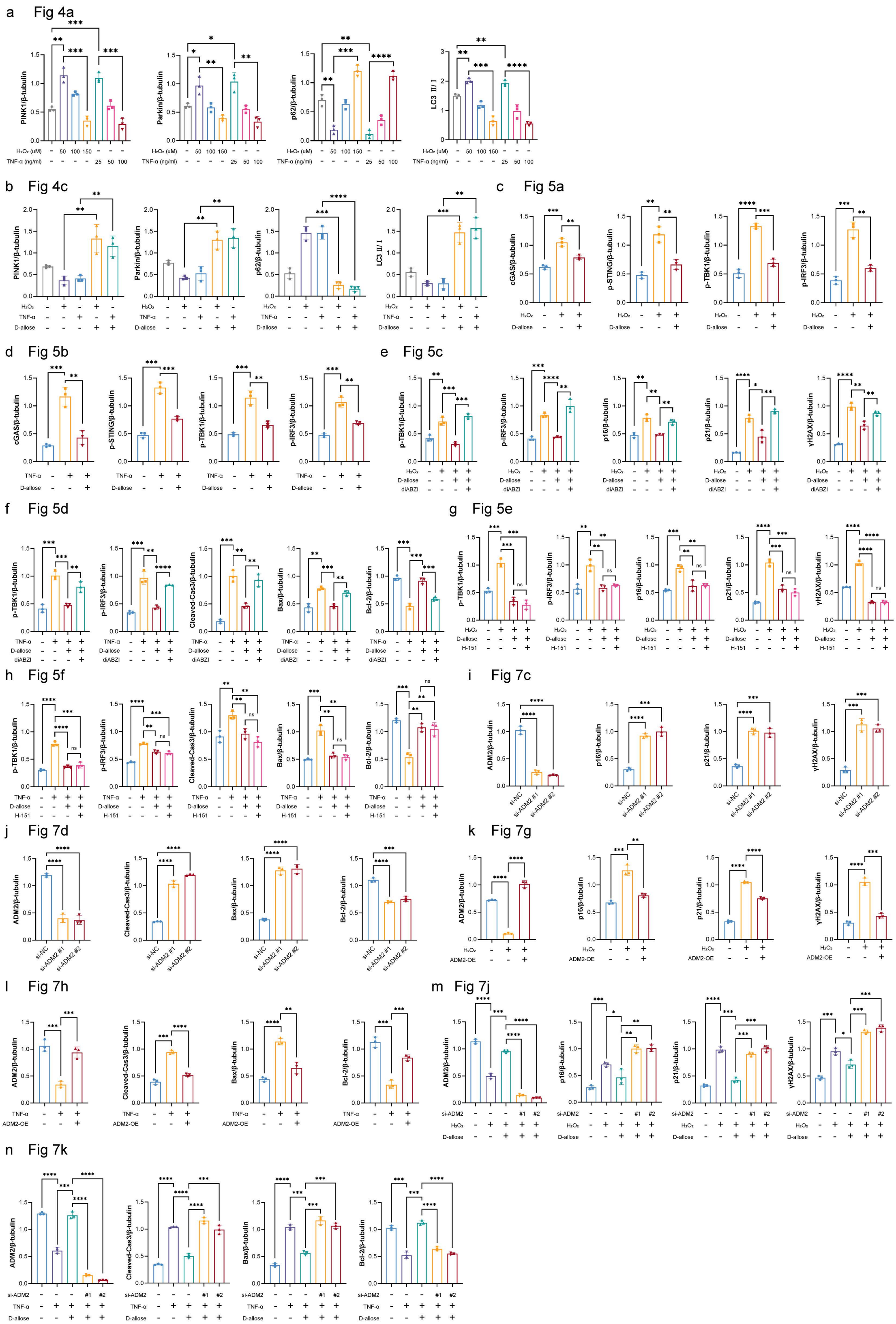

Supplementary Fig. 2 Quantification of western blotting results.

Bar graphs showing the quantification of western blotting results in the corresponding figures.

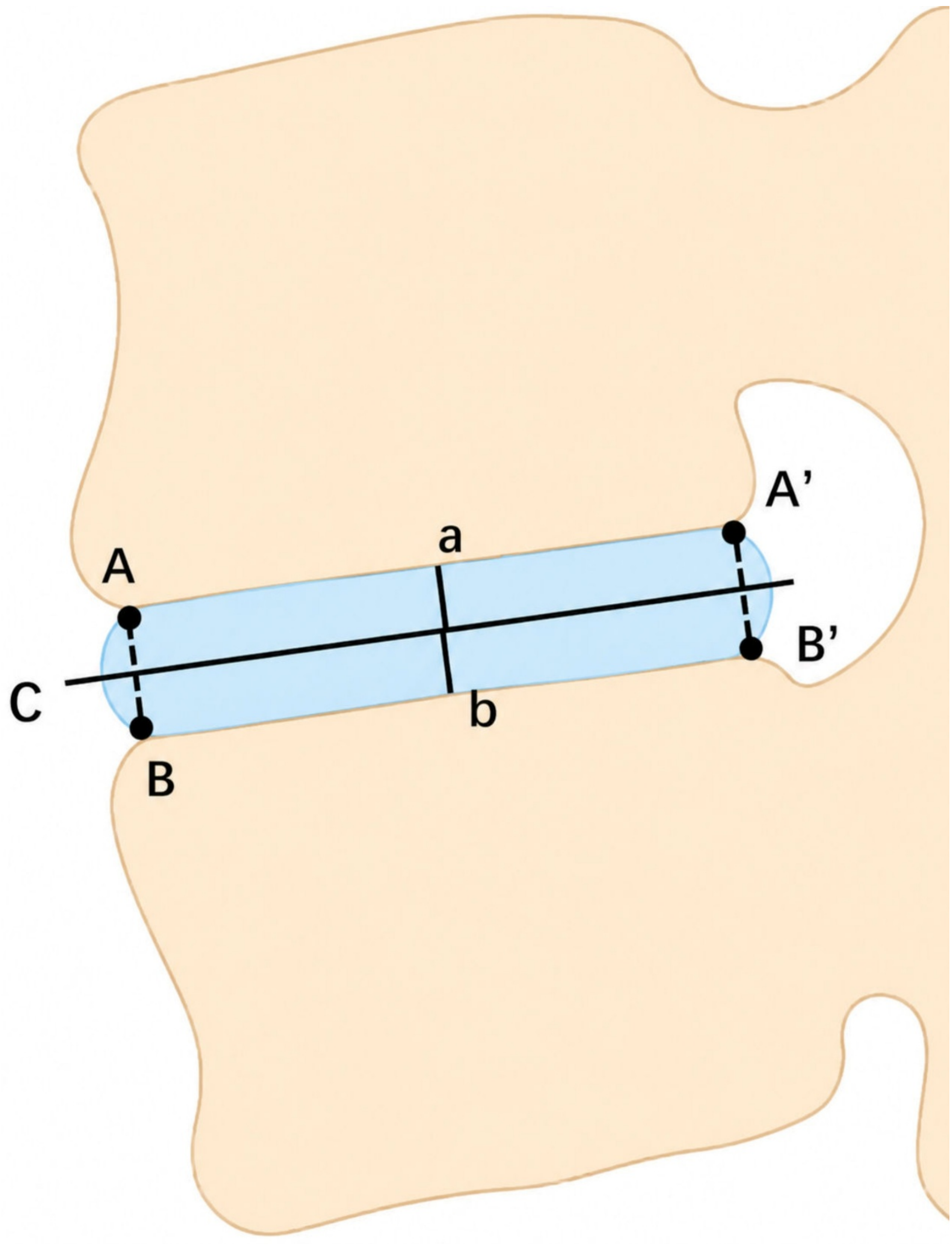

Supplementary Fig. 3 Micro-CT-based disc height measurement

Schematic illustration of disc height measurement on reconstructed micro-CT images.

Supplementary Table 1 primers used in RT-qPCR assay

Primers	Sequences
ADM2	Forward: 5'-GGATGGTGGCCGCCAACACTC
	Reverse: 5'-ACAGGCGGTGGCTGAGATTCTG
MT-Nd1	Forward: 5'-GGCTATATACAACTACGCAAAGGC
	Reverse: 5'-GGTAGATGTGGCGGGTTTTAGG
18s rRNA	Forward: 5’-CTCAACACGGGAAACCTCAC
	Reverse: 5'-CGCTCCACCAACTAAGAACG
GAPDH	Forward: 5'-GGAGCGAGATCCCTCCAAAAT
	Reverse: 5'-GGCTGTTGTCATACTTCTCATGG

Supplementary Table 2 ADM2 siRNA sequence

**Species**	Human
**Symbol**	ADM2
**Catalog No.**	**Hsi123215-a**
**Sequence**	5'- GGACUCAGCUCCUGUGGACTT -3'<br>3'- GUCCACAGGAGCUGAGUCCTT -5'
**Catalog No.**	**Hsi123215-b**
**Sequence**	5'- GCAUCAGCCUCCUCUGCCUTT -3'<br>3'- AGGCAGAGGAGGCUGAUGCTT -5'
**Catalog No.**	**Hsi123215-c**
**Sequence**	5'- CCUGUGGUCUGGAAGCUUCTT -3'<br>3'- GAAGCUUCCAGACCACAGGTT -5'

Supplementary Table 3 Expression profile of ADM2, CHAC1 and NEDD9

gene_id	gene_name	IVDD+D-allose	IVDD	log2FoldChange	pvalue
ENSG00000128165	ADM2	204.1264502	112.5795392	0.858485146	0.000478188
ENSG00000128965	CHAC1	381.3237943	241.9894271	0.656430774	0.000498664
ENSG00000111859	NEDD9	841.254413	1236.718896	-0.555970899	3.72E-08
